# Dissipative Particle Dynamics Modeling of Polyelectrolyte Membrane–Water Interfaces

**DOI:** 10.3390/polym12040907

**Published:** 2020-04-14

**Authors:** Soumyadipta Sengupta, Alexey Lyulin

**Affiliations:** 1 Theory of Polymers and Soft Matter, Department of Applied Physics, Eindhoven University of Technology, 5600 MB Eindhoven, The Netherlands; a.v.lyulin@tue.nl; 2 Center for Computational Energy Research, Department of Applied Physics, Eindhoven University of Technology, 5600 MB Eindhoven, The Netherlands

**Keywords:** interface, dissipative particle dynamics (DPD), polyelectrolyte, cluster, water uptake

## Abstract

Previous experiments of water vapor penetration into polyelectrolyte membrane (PEM) thin films have indicated the influence of the water concentration gradient and polymer chemistry on the interface evolution, which will eventually affect the efficiency of the fuel cell operation. Moreover, PEMs of different side chains have shown differences in water cluster structure and diffusion. The evolution of the interface between water and polyelectrolyte membranes (PEMs), which are used in fuel cells and flow batteries, of three different side-chain lengths has been studied using dissipative particle dynamics (DPD) simulations. Higher and faster water uptake is usually beneficial in the operation of fuel cells and flow batteries. The simulated water uptake increased with the increasing side chain length. In addition, the water uptake was rapid initially and slowed down afterwards, which is in agreement with the experimental observations. The water cluster formation rate was also found to increase with the increasing side-chain length, whereas the water cluster shapes were unaffected. Water diffusion in the membranes, which affects proton mobility in the PEMs, increased with the side-chain length at all distances from the interface. In conclusion, side-chain length was found to have a strong influence on the interface water structure and water penetration rates, which can be harnessed for the better design of PEMs. Since the PEM can undergo cycles of dehydration and rehydration, faster water uptake increases the efficiency of these devices. We show that the longer side chains with backbone structure similar to Nafion should be more suitable for fuel cell/flow battery usage.

## 1. Introduction

**Figure 1:**
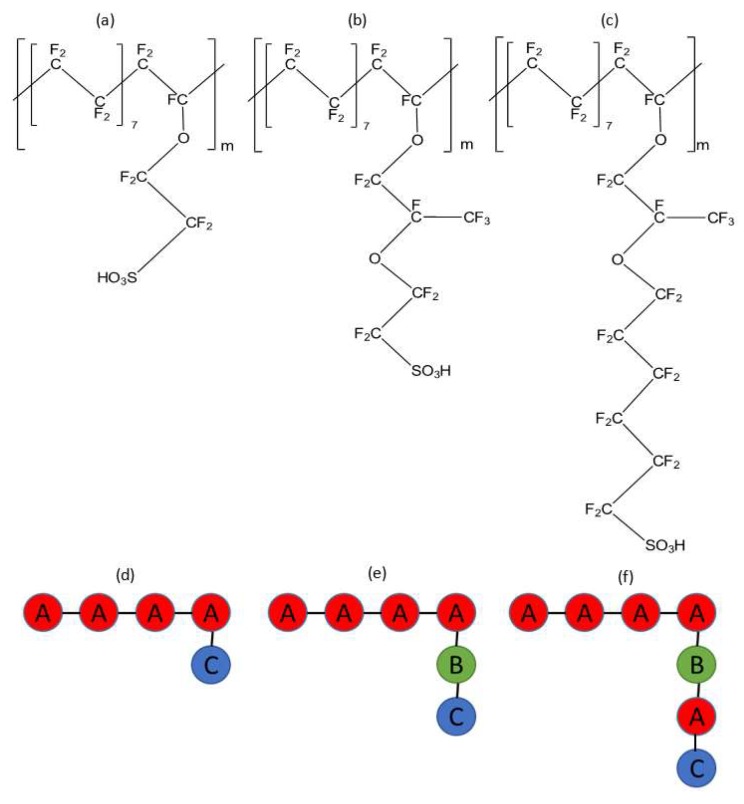
Chemical structures of (**a**) Dow (EW = 980) (**b**) Nafion (EW = 1150), (**c**) Aciplex (EW = 1350), which only differ in side-chain lengths; Bead representation of (**d**) Dow, (**e**) Nafion, and (**f**) Aciplex. Bead A: –CF_2_–CF_2_–CF_2_–CF_2_–, Bead B: –O–CF_2_–CF(CF_3_)–O–, Bead C: –CF_2_–CF_2_–SO_3_H. m = 10 is the simulated polymerization degree. Equivalent weight (EW) is the atomic weight of the monomer divided by the number of ionizable groups per monomer.

Polyelectrolyte membranes (PEMs) are widely used in fuel cells and flow batteries. They are used to provide a pathway for proton transport and separate gases/electrolytes from mixing. PEMs absorb water on contact, and connected water pathways are formed inside the PEM. Polyelectrolyte membranes have a variety of chemical structures. The most commonly used PEM is Nafion, which has a polytetrafluoroethylene (PTFE) hydrophobic backbone and a highly hydrophilic (due to the presence of a sulfonic acid group) side chain; see Figure 1b. A variety of models for the nanomorphology of Nafion have been proposed. Gierke, Hsu, and other co-workers [1,2] suggested water attached to ionic groups, forming spherical clusters of 4 to 6 nm that are distributed in the hydrophobic phase. Electron Microscopy studies found that these clusters were uniformly distributed within Nafion [3]. The side-chain length has been varied to reproduce different types of PEMs. Some of common short side-chain PEMS are Dow (Figure 1a) and Aquivion, both of which have side chains shorter than those in Nafion. Long side-chain PEMs include Aciplex (Figure 1c), which has a side chain longer than in Nafion. The different side-chain chemistry is expected to influence the internal water cluster structure and eventually, the proton diffusion in such PEMs.

There have been various experimental and computational studies to understand the differences induced by varying side-chain lengths on the internal water structure and proton transport in these PEMs. One such experimental study [4] compares the proton conductivities of various PEMs and shows Aquivion (with equivalent weight, EW = 830) at 110 °C having comparable conductivity as Nafion (EW = 1100) at 70 °C across a range of a relative humidities ranging from 50% to 90%. This would imply Aquivion to have lower conductivity than Nafion at the same temperature, which further implies that the side-chain lengths can influence conductivity drastically. Other experimental studies [5] show that the electric conductivity also decreases with increasing EW.

Using classical molecular dynamics (MD) simulations, Allahyarov et al. [6] reported increased diffusion for longer side chains in PEMs of similar EWs. Another classical MD study [7] showed smaller diffusion coefficients as compared to Nafion for both water molecules and hydronium ions in Aciplex, which has a longer side chain compared to Nafion. Sunda et al. [8] showed, using classical MD, an increased diffusion of hydronium ions in Aciplex as compared to Nafion, while it was the opposite for water. They concluded that the increased flexibility of Nafion side chains due to the presence of an extra ether group leads to the increased water diffusion. These contradictory conclusions show that it is very difficult to isolate the effects of the side-chain length from the additional complexities added due to the subtle changes in the side-chain chemistry. However, understanding the individual effects is necessary for the better design of such PEMs.

Ab initio MD simulations have also been used to study and compare PEMs. One such study [9] showed better function group dissociation and stronger water connectivity in a PEM analogous to Nafion as compared to aromatic PEMs. It was concluded that these were the reasons behind the better conductivity of counter ions such as H^+^ and Na^+^ in the Nafion analog PEM measured experimentally. Another ab initio study [10] investigated the effect of different solvents such as water and methanol on the dissociation of functional groups in Nafion, IonClad, and M3 membranes. The functional groups in IonClad and M3 membranes did not dissociate at all in the presence of water, whereas the Nafion functional group dissociated. This was attributed to the presence of the lowering of acidity in IonClad and M3 membranes due to the presence of a benzene ring in the side chain, despite having perfluorinated backbones similar to Nafion. Methanol solvent did not cause any dissociation at all in all three membranes due to the non-polarity of the solvent.

Dissipative particle dynamics (DPD) is a coarse-grained simulation method used to model long-time temporal evolution and large spatial length scales. DPD simulations have been widely used to study PEMs [8,9,10,11,12,13,14,15]. Yamamoto et al. [11] used DPD to analyze the phase-separated Nafion structure and found the simulated structure factors and pore radii close to the experimental values. Wu et al. [12] simulated PEMs of different EWs and found that the water cluster radius increased with decreasing EW, and the opposite was true for the intercluster spacing. Dorenbos et al. [13] reported for a bimodal distribution of side chain spacing that the water diffusion increased with the increasing difference between the two different side chain spacings. It was concluded that a non-uniform spacing of side chains would result in better pore connectivity and water diffusion. Dorenbos [14] found that the water diffusion increased with increasing difference between side-chain lengths for a bimodal distribution of side-chain lengths. Sepehr et al. [15] simulated anion exchange membranes (AEM) using DPD and found water cluster shapes varying with hydration. They also reported larger water domains for chloride anions, as charge carriers, when compared to the hydroxyl anion charge carriers. Ma et al. [16] examined the compatibility and the effect of the varying proportion of components on water diffusion in blend membranes of sulfonated poly ether ether ketone (SPEEK) and poly(vinylidene fluoride)-graft-poly(styrene sulfonated acid) (PVDF-g-PSSA). Komarov et al. [17] used dynamic density functional theory (DDFT), which is also a mesoscale simulation technique similar to DPD, to study the effect of degree of sulfonation and variation in molecular architectures on the water cluster morphology in SPEEK membranes.

Dorenbos and Suga nicely compared the Dow and Nafion membranes in a DPD study [18]. They found that the Nafion membrane with its longer side chain produced more water diffusion as compared to the Dow membrane. It must be noted that all the chemical bonds have the same stretchability and flexibility in the DPD method, as opposed to the classical MD method, where the chain rigidity is controlled by the chemistry specific bond potentials. Therefore, the DPD method is better suited to understand the effect of only the side-chain length variation on internal water clustering and diffusion in such PEMs.

The interface between PEM and water has been studied widely, both experimentally and by some simulations. Zawodzinski et al. [19] concluded that there was a significant resistance to water uptake in Nafion membranes due to the surface hydrophobicity, and such a resistance would influence its performance in fuel cells. Hallinan et al. [20] also found that there was a delay in the initial water uptake for Nafion at low water activity due to the low water concentration gradient. He et al. [21] studied the penetration of D_2_O into the sulfonated polyphenylene (sPP) membranes. They concluded that the polymer interactions with the D_2_O vapor affected the penetration and resulted in a non-uniform distribution of water in the sPP film. They observed different regimes for penetration of D_2_O into the film and also found an excess of D_2_O at the solid silicon interface of the film. One classical MD simulation study reported the sulfonation fraction of PEMs affecting the water/PEM interfacial evolution due to the differences in the hydrophobic and hydrophilic block rearrangement at the interface [22]. All these studies indicate that differences in the side-chain chemistry could have an effect on the penetration of water into the membranes and eventually on the operational efficiency of fuel cells. This motivates our study of the interface of water with PEMs of different side-chain lengths.

A study of the interface between PEM and water offers additional challenges for computational methods such as classical MD. The time scales over which the interfaces evolve are quite large as compared to what can be achieved by classical MD methods. Other methods such as ab initio MD is even more computationally intensive than classical MD, which would make it practically impossible to study the interfacial evolution. The previously mentioned advantages of DPD regarding the non-chemistry specific bond parameters justify its use for this study. In the present paper, using the DPD technique, we have simulated the interface between water and three different PEMs with the same backbone structure but three different side-chain lengths.

## 2. Materials and Methods

### 2.1. Dissipative Particle Dynamics

In the present study, the DPD simulations have been carried out to study the water/PEM interface. As mentioned earlier, the DPD scheme was chosen in order to simulate large time scales over which the interface of such systems evolve. The DPD method was initially developed by Hoggerbrugge and Koelman [23,24] for simulating particle flow with correct hydrodynamics, at a much cheaper computational cost than classical molecular dynamics and lattice gas automata. Later, this method was modified by Groot and Warren [25] by making a connection between the Flory–Huggins χ parameter and the DPD repulsion parameters. This enabled the simulation of various chemical structures using the DPD method [26,27].

Each bead/particle in DPD can be a conglomeration of different atoms. The beads move in space according to Newton’s equations of motion. The force acting on bead *I* is given by

(1) $$F_{i}=\sum_{j\neq i}F_{ij}^{C}+F_{ij}^{D}+F_{ij}^{R}+F_{ij}^{S}$$

where the summation over *j* represents the sum of all forces imparted on bead *i* by all other beads within the cutoff distance *r_c_*°. In Equation (1) $F_{ij}^{C}$ is a conservative force, $F_{ij}^{D}$ is a dissipative force, $F_{ij}^{R}$ is a random force, and $F_{ij}^{S}$ is the spring force.

The conservative force $F_{ij}^{C}$ is defined as

(2) $$F_{ij}^{C}=\{\begin{array}{c} a_{ij}\left(1-\frac{r_{ij}}{r_{c}}\right){\hat{r}}_{ij},\;\;r_{ij}<r_{c}\\ 0,\;\;\;\;\;\;\;\;\;\;\;\;\;\;\;\;\;\;\;\;\;\;\;\;\;\;\;\;r_{ij}>r_{c} \end{array}$$

where $a_{ij}$ is the repulsion parameter between *i* and *j* beads. The repulsion parameter is dependent upon the chemical structure of the beads. $r_{c}$ is the cutoff distance, and it is equal to one DPD length unit.

The pair distance $r_{ij}$

(3) $$r_{ij}=r_{j}-r_{i}\;\;,\;\;\;r_{ij}=\left|r_{ij}\right|$$

is used to define the unit vector ${\hat{r}}_{ij}=\frac{r_{ij}}{r_{ij}}$ along the line connecting the *i* and *j* beads. The dissipative force $F_{ij}^{D}$ is defined as

(4) $$F_{ij}^{D}=-\gamma \omega^{D}\left(r_{ij}\right)\left({\hat{r}}_{ij}\cdot v_{ij}\right){\hat{r}}_{ij},\;\;\;v_{ij}=v_{j}-v_{i}\;\;$$

and it represents the hydrodynamic drag force weighted by the friction factor γ, weighting function $\omega^{D}\left(r_{ij}\right)$, and the component of the velocity vector $v_{ij}$. It acts along the unit vector ${\hat{r}}_{ij}$ connecting *i* and *j* beads. The weighting functions $\omega^{D}\left(r_{ij}\right)$ and $\omega^{R}\left(r_{ij}\right)$ are connected as

(5) $$\omega^{D}\left(r_{ij}\right)={\left[\omega^{R}\left(r_{ij}\right)\right]}^{2}=\{\begin{array}{c} {\left(1-\frac{r_{ij}}{r_{c}}\right)}^{2},\;\;r_{ij}<r_{c}\;\;\\ 0,\;\;\;\;\;\;\;\;\;\;\;\;\;\;\;\;\;\;r_{ij}>r_{c}\; \end{array}.$$

The random force $F_{ij}^{R}$ is defined as

(6) $$F_{ij}^{R}=\sigma \omega^{R}\left(r_{ij}\right)\zeta_{ij}{\left(\Delta t\right)}^{-0.5}{\left(k_{B}T\right)}^{-1}{\hat{r}}_{ij}$$

and it is added to account for thermal fluctuations. $\zeta_{ij}\left(t\right)$ is a random number such that

(7) $$\langle \zeta_{ij}\left(t\right)\rangle =0,\;\;\;\;\langle \zeta_{ij}\left(t\right)\zeta_{kl}\left(t^{\prime }\right)\rangle =\left(\delta_{ik}\delta_{jl}+\delta_{il}\delta_{jk}\right)\delta \left(t-t^{\prime }\right).$$

Equation (7) shows that the random force between a pair of beads is decorrelated with other pairs of beads, as well as in time.

The noise parameter σ and the friction coefficient γ are connected as

(8) $$\sigma^{2}=2\gamma k_{B}T,\;\sigma =3,\;\gamma =4.5,$$

where k_B_ is the Boltzmann constant. The reduced temperature $k_{B}T$ is equal to unity for the simulations. The spring force $F_{ij}^{S}$ acts on beads *i* and *j* connected by a bond. $R_{0}$ is the mean bond length and C is the spring constant. The values of the parameters ($R_{0},\;C,\;\sigma ,\;\gamma )$ are chosen in accordance with previous simulation studies [11,25]

(9) $$F_{ij}^{S}=C\left(r_{ij}-R_{0}\right){\hat{r}}_{ij},\;\;R_{0}=0.86*r_{c},\;\;\;C=50$$

The mass of all beads is equal to unity. The DPD unit of time *τ* = *r*_c_ (*m*/*k_B_T*)^0.5^ corresponds to few tens of picoseconds [11,26] for a typical atomistic system. The cutoff radius *r*_c_ was 0.71 nm, and the beads are propagated by using a modified Verlet integration scheme [25] with a time step Δ*t* = 0.05. The bead density *ρ* is 3, which is the standard value for DPD simulations [25].

### 2.2. Simulation Model

The parameterization used for the different PEMs in this study has also been used in the previous studies of Yamamoto et al. [11] and Dorenbos [28]. Three different type of beads (A, B, and C), i.e., A:–CF_2_–CF_2_–CF_2_–CF_2_–, B:–O–CF_2_–CF(CF_3_)–O–, C:–CF_2_–CF_2_–SO_3_H have been used to construct the polyelectrolyte membranes. Water was modeled as a single bead containing four water molecules following the previous Nafion DPD simulation study [11]. The repulsion parameters ($a_{ij}$) between these beads are shown in Table 1. Beads A and B are primarily hydrophobic and bead C is hydrophilic. Therefore, the repulsion parameter between bead A/B and the water bead (W) is high as compared to the repulsion parameter between beads C and W.

The relation between the repulsion parameter $a_{ij}$ and the Flory–Huggins interaction parameter $\chi_{ij}$ is defined as [25]

(10) $$a_{ij}=a_{ii}+3.27\chi_{ij}$$

The value of $a_{ii}$ is equal to 104 for the parameterization corresponding to a water bead having four water molecules [11]. This value is chosen to match the compressibility of liquid water at room temperature [25]. The $\chi_{ij}$ values were calculated by Yamamoto and Hyodo [11] by using Monte Carlo simulations.

A Nafion chain was constructed by using the A, B, and C beads [11] with the side chain also containing all three types of beads, as shown in Figure 1e. The Dow membrane was constructed using only the A and C beads [28] (Figure 1d). The Dow membrane has a shorter side chain as compared to the side chain of Nafion, and it was composed of only A and C beads. We have also modeled the Aciplex membrane, which has a similar backbone as that of Nafion with a longer side chain, which was modeled using the sequence (A–B–A–C) (Figure 1f). It can be clearly seen that all the PEMs have the same backbone structure but different side-chain lengths (Figure 1d–f). Each chain of the simulated PEMs chains consisted of 10 monomers, following the choice made in the previous molecular dynamics simulation studies [29,30,31].

**Figure 2:**
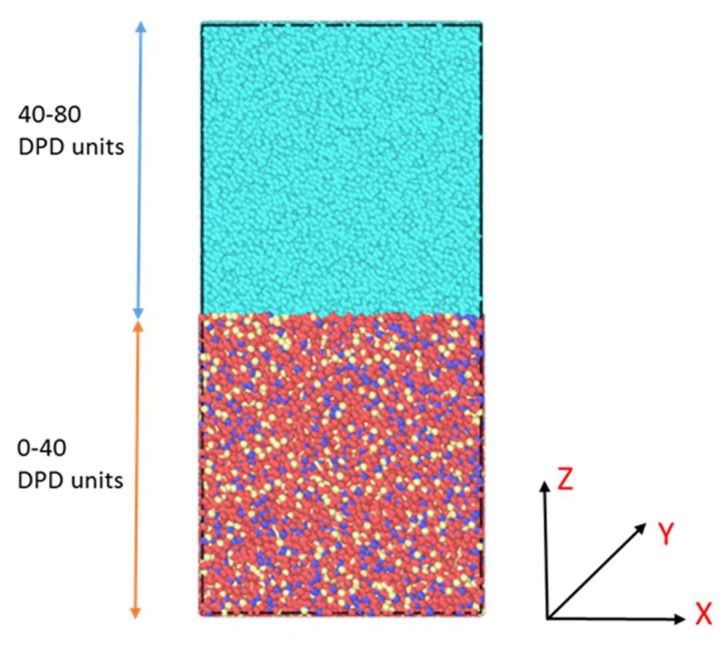
Initial configuration of the simulation box which ranges from 0 to 80 dissipative particle dynamics (DPD) units in the Z-direction. The bottom half of the box, i.e., 0–40 DPD units in the Z-direction are composed of the polyelectrolyte membrane (PEM) beads and the top half of the box, i.e., 40–80 DPD units in the Z-directions are composed of water beads.

The initial configuration for the reported simulations is shown in Figure 2. The bottom half of the simulation box consists of the PEM chains and top half consists of water molecules. The simulation box was constructed at a reduced density [25] of *ρ* = 3. The (X × Y × Z) dimensions were 40 × 40 × 80 DPD units, where the PEM box and the water box were each of the dimension 40 × 40 × 40 DPD units. Therefore, each of the PEM and water simulation boxes had (ρ* times the corresponding volume) 192,000 particles. The number of PEM chains ranged from 2700 to 3800 for the different side-chain length systems. One DPD length unit was equal to $r_{c}=0.71$ nm following the parameterization done in a previous study [11]. Therefore, the simulated system had dimensions of approximately 28 nm × 28 nm × 56 nm in real units.

The water and PEM simulation boxes were constructed separately. Initially, the DPD particles were placed inside the boxes randomly. Reflective fixed boundaries were placed in the Z-direction with periodic boundaries in both X and Y-directions. Thereafter, these boxes were simulated using DPD at a reduced temperature of *T* = 1 for 1 million DPD time steps, to equilibrate the samples. This timescale is much larger than usual timescales used in DPD studies. The chains’ gyration radius had stabilized much earlier (Figure S1), indicating a well-equilibrated sample.

After equilibration of the two separate PEM and water simulation boxes, they were placed on top of each other, as shown in Figure 2. The reflective fixed boundaries were placed in the Z-direction and periodic boundaries were placed in X and Y-directions. Such boundary conditions prevented the interaction between the bottom of the PEM box and the top of the water box and also allowed the interface between PEM and water to evolve. The initial PEM box thickness, 40 DPD units, is more than an order of magnitude higher than the PEM chain radius of gyration (*R*_g_), 1.9–2.1 DPD units (Figure S1), which prevents any geometrical confinement effects to have an effect on the interface evolution. This initial configuration was simulated for 4 × 10^6^ DPD time steps, which is equivalent to a few microseconds of atomistic simulations. Note that the water–PEM interface was still changing after this time. Since our objective was to study the temporal evolution of the interface, we did not simulate for longer.

### 2.3. Analysis Methods

From the simulations, structural and dynamic characteristics such as number density versus distance, Gibbs dividing surface, cluster distribution of water and hydrophilic beads, shapes of clusters of water and hydrophilic beads, and diffusion coefficients of water beads were calculated.

The number density is defined as the number of beads of a particular type in a layer of thickness.

**Figure 3:**
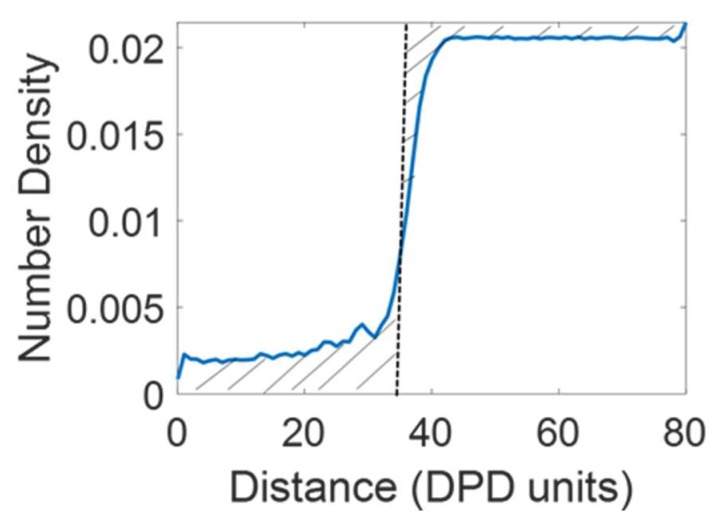
The construction of the Gibbs dividing surface (GDS), dotted black line, for a water number density profile. The shaded areas to the left and to the right of the GDS are equal. Similar procedure was used to calculate the GDS for polyelectrolyte membranes (PEM).

Then, divide one DPD unit (Table 2) at any particular distance from the bottom Z boundary by the total number of beads of the same type in the system. The Gibbs dividing surfaces (GDS) [32], a measure of the interface location, for water and membrane beads were extracted from the number of density profiles. The GDS, as shown in Figure 3, is defined as the distance from the bottom Z boundary at which the shaded areas on either side of the black dotted line are equal. The number of water/membrane beads within the membrane was defined as the number of water (W)/membrane (A, B, C) beads between the GDS_water/membrane_ and the bottom Z boundary.

The simulation box ranging from the bottom boundary up to 45 DPD units in the Z-direction was divided into 20 equal layers (Table 2), with a thickness of 2.25 DPD units, for an analysis of chain statistics. The distance of 45 DPD units was chosen to encompass the interfacial region. The radius of gyration (*R*_g_) of the PEM chains was computed in each of these layers for the entire simulation duration.

The side-chain orientation is defined as the angle between the side-chain vector and the positive Z-axis. The side-chain vector is defined as the vector from the bead connecting the side chain to the backbone to the terminal bead in the side chain. The order parameters (OP) $\langle P_{2}\rangle$, the second-order Legendre polynomials, for the side chain orientations (θ) were also computed in all 20 layers for the entire simulation period.

(11) $$\langle P_{2}\rangle =\frac{3}{2}\langle cos^{2}\theta -1\rangle ,$$

The cluster distribution for water and hydrophilic membrane beads was computed using the OVITO software [33]. A cluster is defined as a group of beads in which each bead is within a pre-defined cutoff distance of at least another bead within that group. The cutoff distance was chosen to be 1.2 DPD units, since this distance was just beyond the W–W RDF and C–C RDF first peaks (Figure S2), and hence would encompass a majority of the respective beads in the cluster formation. For this analysis, the simulation box was divided into three layers comprising of distances 5–15, 15–25, 25–35 DPD units in the Z-direction (Table 2). The upper limit of 35 DPD units was chosen because this distance would cover the interfacial region, since GDS_membrane_ (see further) was in the range of 27–29 DPD units for the different membrane types. The average cluster sizes and the total number of clusters in these three layers were computed for the entire simulation period.

The water cluster shapes were computed for the largest water clusters within 0–26 DPD units in the Z-direction. There was a total of 26 DPD units, which was below the GDS_membrane_ range of 27–29 DPD units for the different membrane types, and hence, would comprise only the membrane. The eigenvalues ($\lambda_{x}$,$\;\lambda_{y}$,$\;\lambda_{z})$ that characterize the water cluster shapes were extracted from the gyration tensor of the largest water cluster where $\lambda_{z}\geq \lambda_{y}\geq \lambda_{x}$. The quantities such as acylindricity, asphericity, and relative shape anisotropy (RSA) as shown in Equations (12)–(14) were computed from the eigenvalues and compared for the different membrane types through the simulated period.

(12) $$Asphericity=\lambda_{z}^{2}-0.5\left(\lambda_{x}^{2}+\lambda_{y}^{2}\right)$$

(13) $$Acylindricity=\lambda_{y}^{2}-\lambda_{x}^{2}$$

(14) $$RSA=1.5\left(\frac{\lambda_{x}^{4}+\lambda_{y}^{4}+\lambda_{z}^{4}}{{\left(\lambda_{x}^{2}+\lambda_{y}^{2}+\lambda_{z}^{2}\right)}^{2}}\right)-0.5$$

We stress that in the present simulations, the samples never achieved steady state. However, the GDS changes very minutely over the large periods of time, as we have discussed later. In addition, the simulation time in the present study is of a few microseconds, which is orders of magnitude higher than the characteristic relaxation time for water. We analyzed the mean square displacements (MSD) of the water beads for the different membrane types in the last 1 million DPD time steps in four different layers of thickness 8 DPD units (Table 2) starting from the bottom boundary.

## 3. Results

### 3.1. Interface Evolution

**Figure 4:**
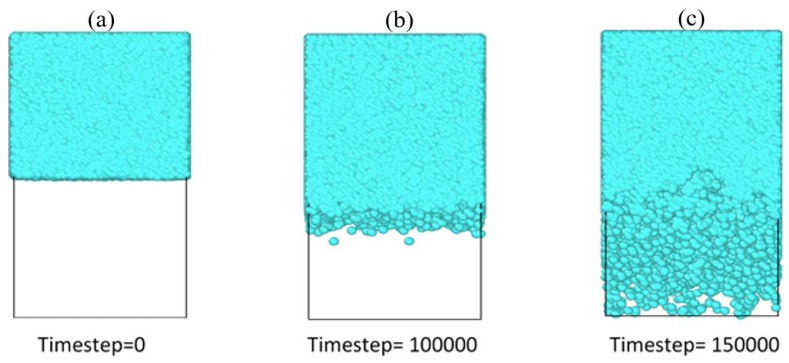
Evolution of the water–polyelectrolyte membranes (PEM) interface for Aciplex (**a**) At time step = 0, all water is present in the top half of the box. The bottom half of the box is the membrane, which has not been shown here to clearly see the water interface. (**b**) At time step = 100,000, water has penetrated some distance into the membrane, with some isolated clusters formed. (**c**) Water has penetrated almost completely into the membrane, with a more homogeneous distribution of the water clusters.

Figure 4 shows the typical snapshots with the time evolution of the water–PEM interface. Only water beads have been shown for more clarity. We can see that water is quite continuous near the initial interface at time step = 100,000. Some isolated clusters have formed near the maximum depth up to which water has penetrated the membrane. Overall, the clusters inside the membrane are in the shape of a thin film. With time (see time step = 150000), water has penetrated almost the entire membrane, and the clusters have distributed more homogeneously. We can see the clusters are more isolated near the bottom of the membrane and become more continuous on proceeding toward the initial interface. We have provided later (see the discussion after Figure 9) a more quantitative discussion on the evolution of the shape of these water clusters.

**Figure 5:**
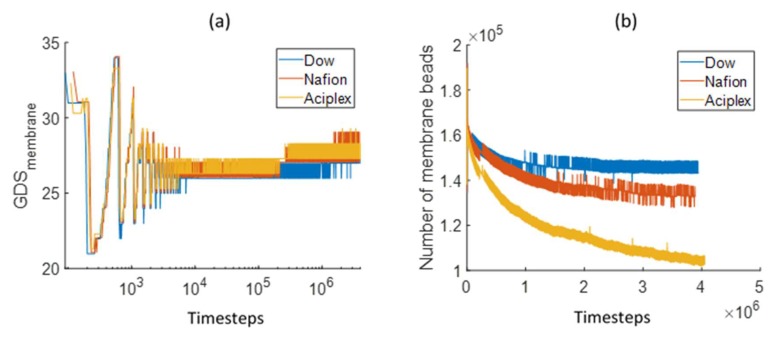
Comparison of (**a**) the temporal evolution of the membrane Gibbs dividing surface (GDS) and (**b**) time dependence of the number of membrane beads between the GDS membrane and bottom Z boundary for different side-chain length PEMs.

The GDS was extracted from the density profiles, both for the membrane and for water beads. Figure 5a shows the comparison for the GDS_membrane_ temporal evolution for different side-chain length PEMs. Initially, the GDS position fluctuates largely for all the three different membrane types. The GDS is relatively stabilized after around 10,000 time steps. On closer inspection, it can be noticed that the GDS slowly increases after this time. In addition, the GDS position increases more with increasing side chain length. Figure 5b shows the number of polymer beads (A+B+C) within the membrane as per the procedure described previously. This diagram provides a clearer picture of the swelling of the membrane. The amount of polymer beads within the membrane decreases with the increasing side chain length. Aciplex shows the maximum decrease followed by Nafion and Dow. This observation indicates that water is filling up the Aciplex membrane fastest, followed by Nafion and Dow membranes; such a swelling leads to the observed changes in the corresponding GDS locations. This also means that the membrane swelling rate is proportional to the side-chain length.

**Figure 6:**
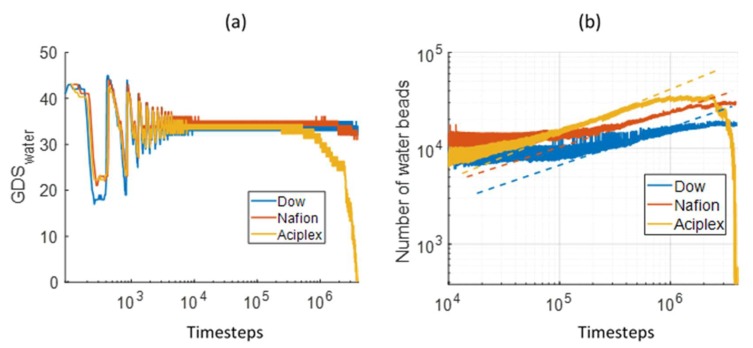
Comparison of (**a**) the temporal evolution of the Gibbs dividing surface (GDS) for water for different side-chain length PEMs, and (**b**) time dependence of the number of water beads between GDSwater and bottom Z boundary for different side-chain length PEMs. The yellow dotted line in **(b**) has a slope of 0.5, and the orange and blue dotted lines in (**b**) have slopes slightly less than 0.5.

The water interface is also evolving along with the membrane interface. Figure 6a shows the GDS_water_ position for different PEMs. These plots also concur with the previous conclusion about the swelling rate dependency on the side-chain length. The GDS_water_ position also fluctuates hugely at very small times, after which it starts decreasing slowly. The GDS location decreases with increasing side-chain length. In fact, the GDS_water_ for the long side-chains Aciplex rapidly approaches and crosses the bottom Z boundary due to the very fast diffusion of water. Figure 6b shows the number of water beads between the GDS and the bottom Z boundary, which can serve as a representation of the water uptake of the membrane. It can be seen that the water uptake for the simulated time increased with the increasing side chain length. This has also been observed experimentally where Nafion showed more water uptake than a shorter side-chain Aquivion membrane [4]. It is interesting to add here that He et al. [21] had found two different regimes for the penetration of D_2_O into the sulfonated polyphenylene (sPP) membranes. Initially, the water uptake rate scaled with $t^{0.5}$ and reduced at a later time. In the present simulations, we do see an initial regime where the water uptake increases rapidly and then starts slowing down. The slope of this initial regime is approximately 0.5 for Aciplex and is only slightly smaller than 0.5 for Nafion and Dow. We also observed that the starting times for this rapid increase regime decreases with increasing the side-chain lengths. The slope of 0.5 can be explained by a purely diffusive motion of water molecules getting adsorbed on the PEM. With increasing time, the diffusion of water is obstructed due to the formation of more tortuous pathways, which is reflected in the reduced slope of the water uptake plot.

### 3.2. Chain Statistical Properties

**Figure 7:**
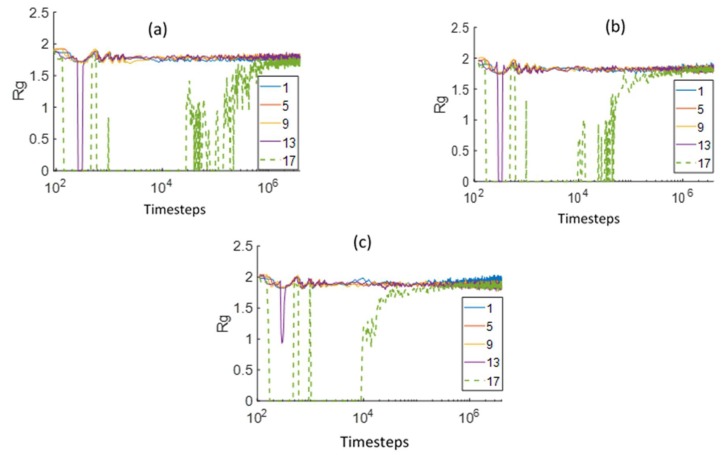
Comparison of the chain radius of gyration (*R*_g_) in different layers (1–17), at varying distances from the bottom Z boundary, for (**a**) Dow, (**b**) Nafion, and (**c**) Aciplex PEMs. Layer 1 is nearest to the bottom Z-boundary and layer 17 is at the interface of water and the membrane. Each layer is 2.25 DPD units thick. The green dashed line is the Rg value in a layer near the water–PEM interface.

The PEM chain radius of gyration (*R*_g_) was computed in 20 layers, each with a thickness of 2.25 DPD units, at different distances from the bottom boundary and is shown in Figure 7. The layer 1 is adjacent to the bottom Z boundary and the layer numbers increase on moving upwards. The layers 1, 5, 9, and 13 will be referred to as the membrane layers, and layer 17 will be referred to as the interfacial layer due to its proximity to the initial water PEM interface. The chain was considered to be in a layer if the center of mass of the chain was present in the particular layer. The membrane layers (layers 1, 5, 9, and 13) initially contain only the membrane beads. As clearly seen from the results in Figure 7, the membrane layer *R*_g_ values for the different side-chain lengths show a similar pattern with time. At very small times (approximately 10^2^ DPD time steps), there is a slight decrease of *R*_g_ in these layers due to the influx of water, since the primarily hydrophobic chains start to shrink. After this initial period, the *R*_g_ reaches a stable value in the membrane layers. The *R*_g_ value in the interfacial layer (layer 17) decreases rapidly at the small times (approximately 10^2^ DPD time steps) due to the presence of excessive water near the interface. After around 10^4^ DPD time steps, the interfacial layer *R*_g_ starts increasing due to swelling of the membrane. The *R*_g_ value in the interfacial layer eventually stabilizes at a value equal to that of the membrane layers.

A clear difference in the times at which the interfacial layer *R*_g_ starts increasing can be observed for the different PEMs. This time increases with the decreasing side chain length. In addition, the rate of increase in *R*_g_ increases with the side-chain length. The Aciplex PEM interfacial layer *R*_g_ starts increasing earliest and increases rapidly without much oscillations to stabilize at a final value. Whereas, Dow PEM with the shortest side chain shows a very slow increase in *R*_g_ with many oscillations. These observations also show that the rate of swelling of the membranes is proportional to the side-chain length.

**Figure 8:**
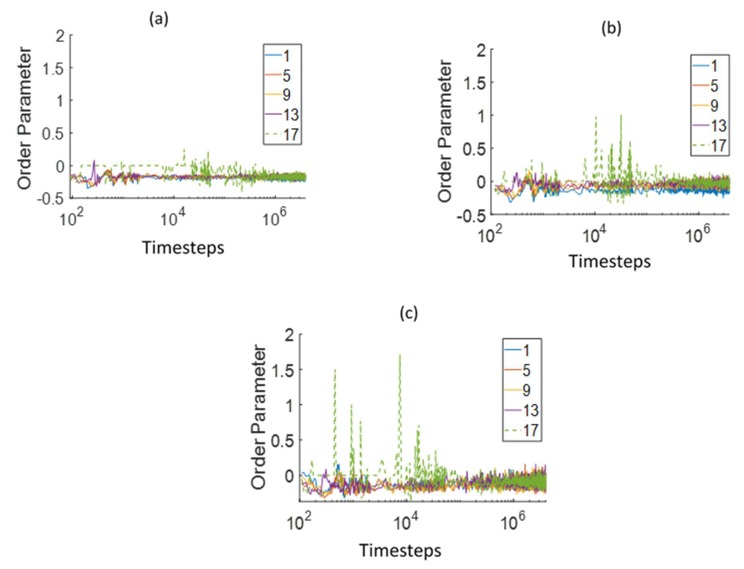
Comparison of the side-chain order parameter (OP) with respect to the Z-axis in different layers, at varying distances from the bottom Z boundary, for (**a**) Dow, (**b**) Nafion, and (**c**) Aciplex PEMs. Layer 1 is nearest to the bottom Z boundary and layer 17 is at the interface of water and the membrane. Each layer is 2.25 DPD units thick. The green dashed line is the OP in a layer near the water–PEM interface.

Figure 8 shows the side-chain order parameter (OP) computed with respect to the Z-axis in the same layers as used for the *R*_g_ analysis. The side chain was considered to be in a layer if the center of mass of the side chain was present in the particular layer. The interfacial layer (layer 17, Figure 8) OP was slightly higher than that of the membrane layers for small times (up to 10^4^ DPD time steps) for the different side-chain lengths. This could be due to the largely water-filled space initially in the interfacial layer, which allowed the slightly hydrophilic side chain to orient along the primary water diffusion direction. It can also be seen during this time that the OPs in the interfacial layer were visibly higher for the PEM with longer side chains. This implies that the side chains tended to align with the water flow direction more in the interfacial layers for the PEMs with the longer side chains. However, at large times, the OPs in all the layers stabilized at the same value.

### 3.3. Water Cluster Formation Dynamics and Morphology

Water present in the PEMs agglomerate to form big clusters which aid in proton transport. The side chains orient preferably along the water clusters because of the hydrophilic tip of the side chains. Therefore, it is important to study the dynamics of the water clusters formation at the interface for different side-chain lengths.

**Figure 9:**
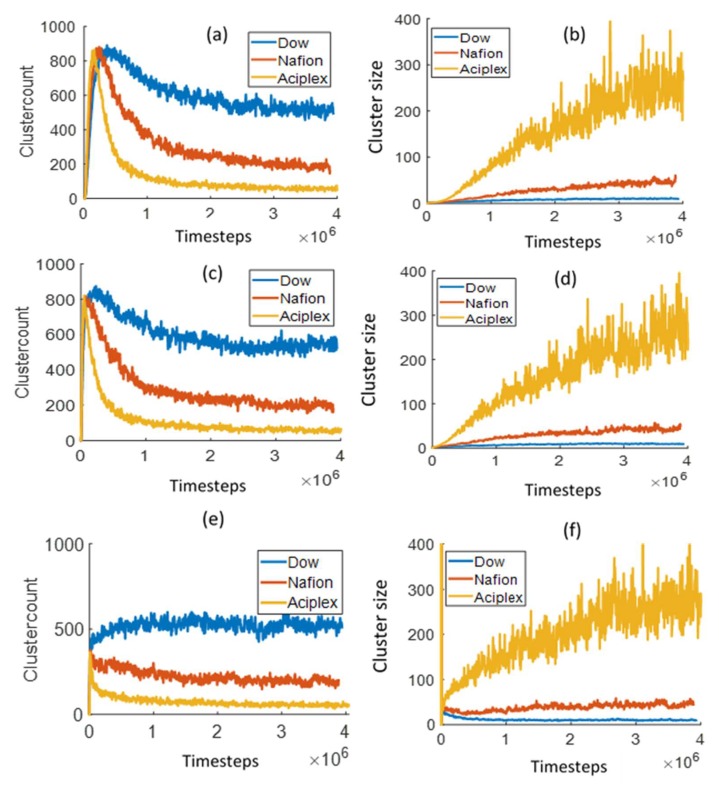
Comparison of the time evolution of water cluster count/ number of clusters and average cluster size for different side chain length PEMs in three different layers at distances ranging from (**a**,**b**) 5–15 DPD units (**c**,**d**) 15–25 DPD units and (**e**,**f**) 25–35 DPD units from the bottom Z boundary.

Figure 9 shows the total number of water clusters and the average cluster size in three different layers of thickness 10 DPD units each and at a distance of 5, 15, and 25 DPD units from the bottom Z boundary. These layers will be referred to as the bottom, mid, and top layer for the analysis of cluster size and number of clusters/cluster count. In the bottom and mid layers, the water cluster count peaks after a certain (rather small) time and then starts decreasing slowly for the different side-chain length PEMs. The time to peak can be interpreted as the time after which the water beads start agglomerating into bigger clusters due to lack of free space. The time to peak and the number of clusters at large times are inversely proportional to the side-chain lengths. The larger number of clusters indicates a more dispersed water phase. The observations from the time dependence of clusters number show that the water diffusion rate into the membrane is proportional to the side-chain length. The water cluster sizes also increase with time in the mid and bottom layers, and the average cluster sizes are noticeably higher for increasing side-chain lengths. In the top layer, which consists of the interface, the cluster count goes down for the two largest side-chain length PEMs, while it increases for the smallest side-chain length PEM. This would be due to the small rate of water diffusion in the Dow membrane, which allows the water phase to stay dispersed even near the interface for a longer time period. The water cluster size increases for Aciplex and Nafion in the top layer, while it almost stays constant for the Dow membrane. This too confirms the slow formation of a connected water phase for the Dow membrane, even at distances close to the interface.

The hydrophilic beads present in the side chain disperse along the water clusters. Therefore, the analysis of the cluster evolution of these hydrophilic beads will throw more light on the water cluster formation dynamics. This is discussed in detail in the Supplementary Material and Figure S3.

**Figure 10:**
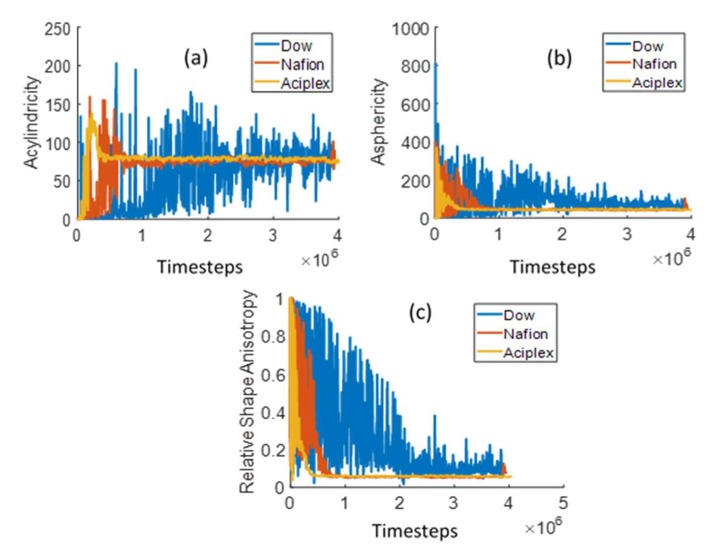
Time evolution of the largest water cluster (**a**) acylindricity, (**b**) asphericity, and (**c**) relative shape anisotropy (RSA) in the 0–26 DPD units range in the Z-direction.

We have also studied the time evolution of the shape of the water clusters with time for the different side-chain length PEMs. The procedure for extracting the shape characteristics have been explained before in the Analysis Methods section. Figure 10 shows three parameters, i.e., acylindricity, asphericity, and relative shape anisotropy (RSA) for the different PEMs studied. Both acylindricity and asphericity go up initially and then come down and stabilize for all the three different side chain lengths. The final stabilization value is the same for all the three PEMs. This implies that the side-chain length does not have any effect on the final water cluster structure shapes. A possible reason behind this could be that the same structure of hydrophobic backbones for all three PEMs form similar shaped hydrophobic domains and consequently hydrophilic domains. Only the time to peak and the time to relax to a stable value is inversely proportional to the side-chain lengths. This is to be expected, since the water penetration and hydrophilic domain formation increases with the side-chain lengths, as mentioned previously. The RSA value (Figure 10c) is initially close to unity for all three PEMs. This implies that the initial water clusters are in the form of narrow tubes. The RSA value decreases rapidly toward 0.2 for all the three membranes. Interestingly, RSA = 0.25 implies a thin film of water as calculated from Equation (14), with $\lambda_{z}=\lambda_{y}\;and\;\lambda_{x}=0$. This thin water film near the interface can also be seen in Figure 4b for Aciplex at time step =100,000. With time, the RSA decreases relatively slowly from around 0.2 to stabilize at a value of around 0.08, which is indicative of a spherical cluster, for all the PEMs. We can see the same progression toward a more spherical shape in Figure 4c where the clusters become more homogeneously distributed through the membrane. In addition, the same final stabilization value of RSA for all the different PEMs also suggests no influence on the final shapes of water clusters due to the side-chain lengths.

### 3.4. Water Diffusion

**Figure 11:**
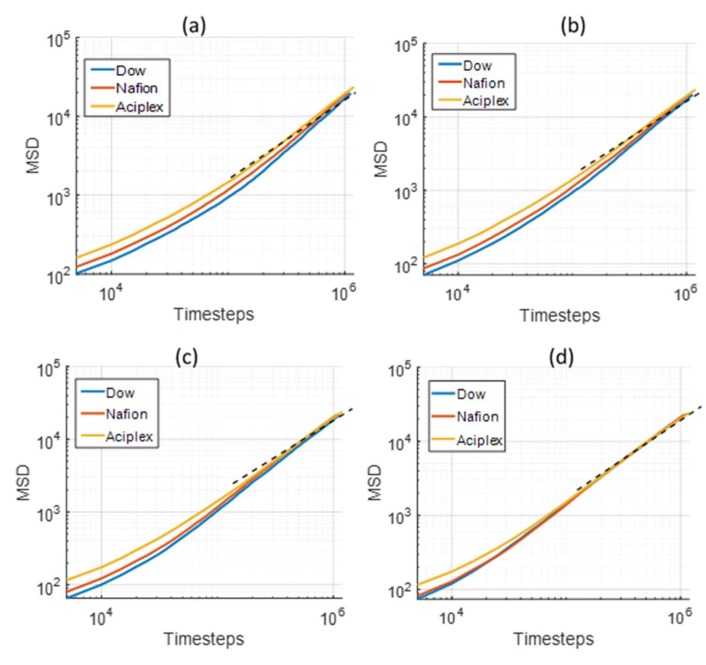
Comparison of the mean square displacement (MSD) of water for different side-chain length PEMs in four different layers at distances ranging from (**a**) 0–8 DPD units, (**b**) 8–16 DPD units, (**c**) 16–24 DPD units, and (**d**) 24–32 DPD units from the bottom Z boundary. The dotted line shows the unit slope.

As observed from the GDS evolution, the system never reaches a steady state. However, the changes in GDS positions at very large times (>3 × 10^6^ time steps) are negligible. Therefore, we decided to analysze the mean square displacements (MSDs) of the water beads for the last 1 million time steps in four different layers of thickness for 8 DPD units, starting from the bottom Z boundary, as shown in Figure 11. The rate of water diffusion is directly proportional to the change in MSD as per the Einstein relation. The MSD increased with the side-chain lengths, which means that the rate of water diffusion was maximum for Aciplex followed by Nafion and Dow, respectively, in all the different layers. This observation agrees with our previous analysis, which shows that the water penetration rate into the PEMs is directly dependent on the side-chain lengths. The difference between the MSDs for the different PEMs continuously decreases from the bottommost layer to the topmost layer. This is because the water in the topmost layer is present almost in bulk form and hence diffuses independent of the effects of the PEMs.

## 4. Conclusions

The water interface of three different PEMs materials, Dow, Nafion, and Aciplex, with different side-chain lengths, were simulated using DPD technique. The number of water beads within the polyelectrolyte membrane increased with increasing the PEM side-chain lengths, implying the increased swelling rate. A rapidly increasing swelling regime followed by a slowing down was observed in the swelling patterns. A slope of 0.5 was found for this rapid swelling regime, which agrees qualitatively with the previous experimental observations [21]. The start of the rapidly increasing regime was observed to be delayed with decreasing the side-chain length.

The rate of increase of the chain radius of gyration near the interface was also proportional to the side-chain lengths. Initially, the longer side chains were slightly more aligned toward the bulk water slab, indicating that the longer side chains reacted more easily to water flow.

The average water cluster size and the rate of its increase at different distances from the interface increased with the side-chain length. The number of clusters formed by the PEMs side-chain hydrophilic beads increased with the influx of water, indicating the dispersion of the side chains along the water clusters. In addition, the rate of increase of the number of clusters is becoming larger for long side chains, indicating faster rearrangement of the corresponding polymer chains. With time, the water clusters becoming more homogeneously distributed through the membrane, and their relative shape anisotropy decreased, which indicates a spherical shape of these clusters.

The water diffusion into the membrane increased on approaching closer to the interface. In addition, the water diffusion was found to be proportional to the side-chain lengths.

The water uptake rate is an important factor in the operation of fuel cells. The Aciplex membrane with the longest side-chain length showed the fastest water uptake. Faster water uptake can increase the efficiency of these devices, since the PEM can undergo cycles of dehydration and rehydration during operation. Therefore, longer side chains with a backbone structure similar to Nafion should be more suitable for fuel cell or flow battery usage.

## Figures and Tables

**Table 1:** Flory–Huggins interaction parameter and dissipative particle dynamics (DPD) repulsion parameter for different bead pair.

Bead Pair	$\chi_{ij}$ (Flory–Huggins Parameter)	$a_{ij}$ (DPD Repulsion Parameter)
A–B	0.022	104.1
A–C	3.11	114.2
A–W	5.79	122.9
B–C	1.37	108.5
B–W	4.90	120.0
C–W	−2.79	94.9

**Table 2:** Number and location of layers for different analyses carried out in the study.

Analysis	Number and Location of Layers
Number density	80 layers of 1 DPD unit thickness in the Z-direction
Chain radius of gyration (*R*_g_) and side-chain order parameter (OP)	20 layers of 2.25 DPD unit thickness starting from the bottom Z boundary
Cluster analysis	3 layers of thickness of 10 DPD units starting at 5, 15, and 25 DPD units distance from the bottom Z boundary
Mean square displacement (MSD)	4 layers of 8 DPD unit thickness starting from the bottom Z boundary

## Supplementary Materials

The following are available online at https://www.mdpi.com/2073-4360/12/4/907/s1, Figure S1: Comparison of radius of gyration (*R*_g_) for the dry Dow, Nafion and Aciplex membranes, Figure S2: Comparison of radial distribution function (RDF) for the hydrated PEMs for (a) C–C (b) W–W at 2 × 10^6^ time steps, Figure S3: Comparison of the time evolution of the hydrophilic bead cluster count/number of clusters and average cluster size for different side-chain length PEMs in three different layers at distances ranging from (a),(b) 5–15 DPD units (c),(d) 15–25 DPD units, (e),(f) 25–35 DPD units from the bottom Z boundary.
