# Supplementary material for: Dissipative Particle Dynamics Modeling of Polyelectrolyte Membrane–Water Interfaces
Source: Polymers (Basel). 2020 Apr 14;12(4):907. doi: 10.3390/polym12040907 (PMC7240515; doi:10.3390/polym12040907)
Supplement: Supplementary file 1 [file polymers-12-00907-s001.zip › polymers-748376-supplementary.docx]

Dissipative Particle Dynamics Modelling of Polyelectrolyte Membrane-Water Interfaces

Soumyadipta Sengupta and Alexey Lyulin

S1. Radius of Gyration


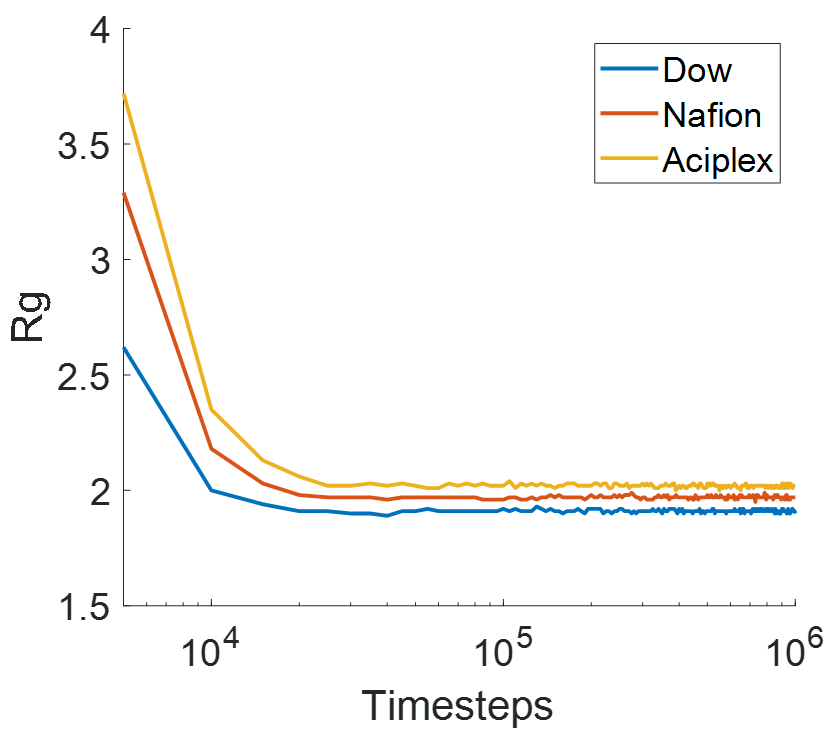


**Figure S1.** The comparison of the gyration radius (*R_g_*) for the dry Dow, Nafion and Aciplex membranes.

Figure S1 shows the radius of gyration (*R_g_*) for the dry PEMs during the initial 1 million DPD timesteps. The *R_g_* values have stabilized well before this time.

S2. Radial Distribution Function


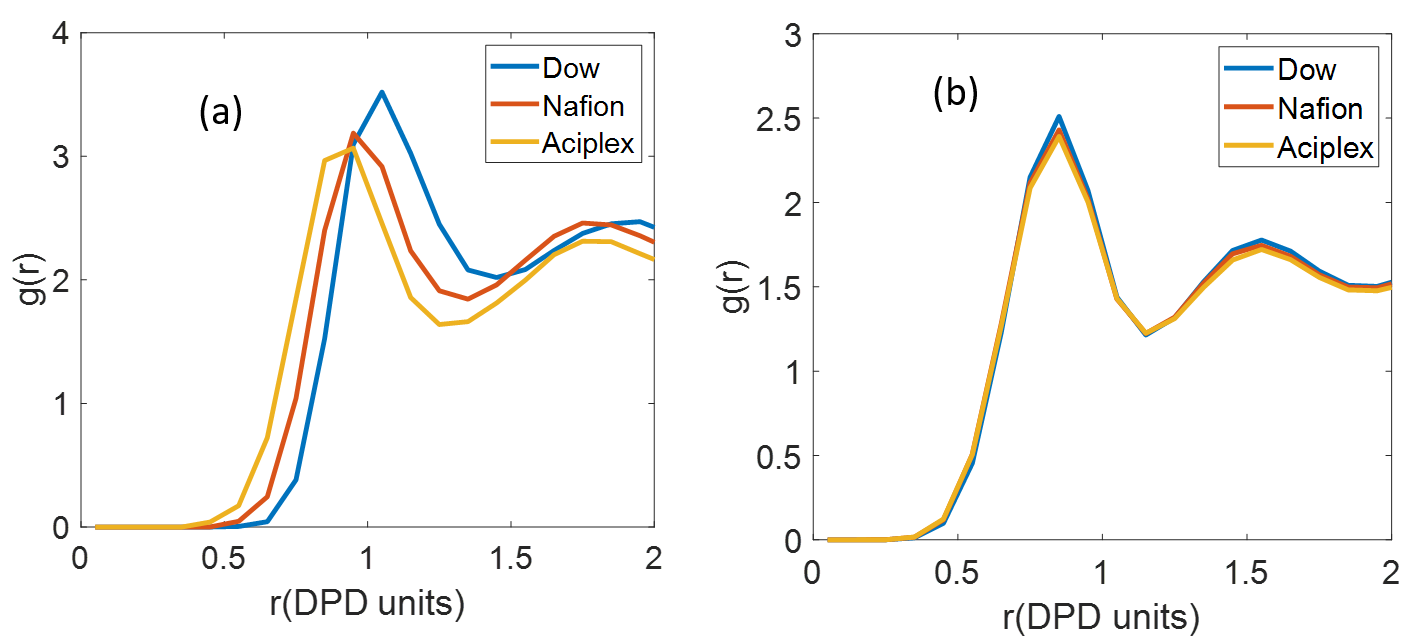


**Figure S2.** The comparison of the radial distribution functions (RDF) for the hydrated PEMs for (**a**) C–C (**b**) W–W pairs after 2 × 10^6^ timesteps.

Figure S2a shows the comparison of the RDFs for the hydrophilic beads (C–C) and Figure S2b shows the comparison of RDFs for the water beads (W–W).

S3. Hydrophilic Bead Cluster Formation Dynamics


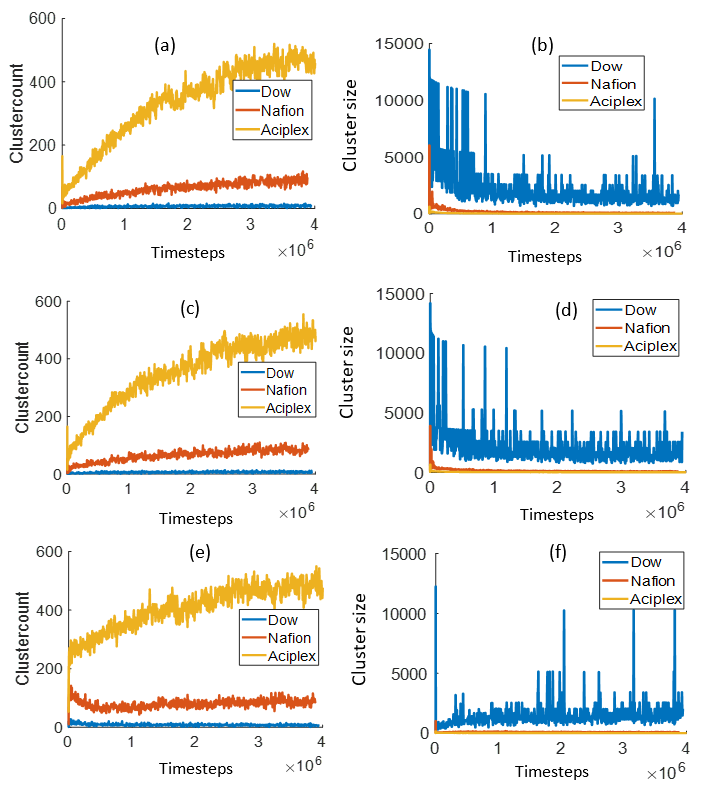


**Figure S3.** Comparison of the time evolution of the hydrophilic bead clustercount/number of clusters and average cluster size for different side chain length PEMs in three different layers at distances ranging from (**a**,**b**) 5-15 DPD units (**c**,**d**) 15-25 DPD units (**e**,**f**) 25–35 DPD units from the bottom Z boundary.

The hydrophilic bead cluster count goes up for the three different membranes in the bottom and mid layers along with a decrease in the cluster sizes. This indicates that a rearrangement of the side chains is taking place and the hydrophilic beads, at the end of the side chains, are getting more dispersed in these layers. The number of clusters increases more with increasing the side chain length, and the average cluster sizes decrease more with increasing the side-chain length. This implies that the rate of therearrangement of the hydrophilic tips of the side chains is proportional to the side-chain length. This could be because the longer side chains can penetrate deeper into the water domains away from the hydrophobic domains, thus allowing easier movement of the hydrophilic tip of the side chains. In the top/interfacial layer, the sulfur cluster count increases for the Aciplex PEM and goes down for the other two shorter side-chain PEMs. The cluster sizes increase noticeably for Dow, but remain almost constant for the other two PEMs. This is due to the very slow penetration of water into the Dow membrane which leads to slow dispersion and rearrangement of sulfur clusters at the interface as compared to the other two PEMs.
